# Pulsed Electric Field (PEF) Processing of Chilled and Frozen-Thawed Lamb Meat Cuts: Relationships between Sensory Characteristics and Chemical Composition of Meat

**DOI:** 10.3390/foods10051148

**Published:** 2021-05-20

**Authors:** Kevin Kantono, Nazimah Hamid, Diksha Chadha, Qianli Ma, Indrawati Oey, Mustafa M. Farouk

**Affiliations:** 1 Department of Food Science, Faculty of Health and Environment Sciences, Auckland University of Technology, Auckland 1010, New Zealand; kkantono@aut.ac.nz (K.K.); diksha.chadha@aut.ac.nz (D.C.); Maql1@newhope.cn (Q.M.); 2 Key Laboratory of Agro-Products Processing, Institute of Food Science and Technology, Chinese Academy of Agricultural Sciences, Ministry of Agriculture and Rural Affairs, Beijing 100193, China; 3 Department of Food Science, University of Otago, PO Box 56, Dunedin 9054, New Zealand; indrawati.oey@otago.ac.nz; 4 Riddet Institute, Massey University, Palmerston North 4472, New Zealand; 5 AgResearch MIRINZ, Ruakura Research Centre, Private Bag 3123, Hamilton 3240, New Zealand; mustafa.farouk@agresearch.co.nz

**Keywords:** pulsed electric field, lamb, sensory, temporal dominance of sensations, cuts, storage, meat

## Abstract

The effect of PEF processing and chilled storage on the volatile composition and sensory properties of chilled and frozen lamb cuts was investigated in this study. Results showed that PEF-treated chilled and frozen lamb cuts varied in temporal flavour attributes with storage. Storage for 7 days resulted in oxidized flavour, while PEF treatments for all chilled and frozen cuts were associated with browned and livery flavour attributes. Partial least squares regression (PLSR) was applied to determine the predictive relationships between the volatile composition, fatty acid and amino acid profiles, and sensory responses for PEF treated lamb cuts. The results showed that some volatile compounds (2-nonanone, 2-pentylfuran, pyrrole, methyl pyrazine, 2-ethyl-3-methyl pyrazine, and thiophene) correlated well with the meaty and juicy flavour of PEF treated frozen lamb cuts. In PEF treated chilled lamb cuts, meaty and juicy flavours were associated with the presence of fatty acids (C18:0, SFA, 20:5(n-3), and n-3). In contrast, livery and browned perception of both PEF processed chilled and frozen lamb cuts were associated with the presence of amino acids (threonine, phenylalanine, isoleucine, tyrosine, and methionine), and some volatile compounds (heptanal, 2-ethylfuran, pyridine, dimethyl disulphide, dimethyl trisulphide, and 3,5-diethyl-2-methyl pyrazine). Overall, these results imply that careful consideration of type of meat cuts, PEF pre-treatment, and storage are important when subjecting lamb meat to PEF processing.

## 1. Introduction

Pulsed electric field (PEF) is a novel non-thermal technology that utilises short pulses of electricity for microbial inactivation and has been widely researched recently in the processing of solid food such as meat [1,2,3,4]. PEF can induce changes in the physical properties of meat such as structure and texture. PEF treatment has been reported to improve tenderness in *M. semitendinosus* beef [3,4]. *M. semimembranosus and M. Longissimus lumborum* beef [2], and turkey breast meat [1]. The application of PEF in meat (especially beef) can enhance cell permeability due to electroporation and consequently enhance proteolysis that contributes to tenderisation [2,3].

Although PEF has a positive impact on meat tenderisation, undesirable effects on lipid oxidation have been reported. The generation of free radicals and hydroperoxides from unsaturated fatty acids due to disruption to cell membranes can further result in secondary products, which are responsible for off-flavours and odours that can adversely affect meat eating quality. Arroyo, Eslami, Brunton, Arimi, Noci and Lyng [1] reported no influence of PEF treatment (1.1–3 kV/cm) on lipid oxidation of turkey meat after 5 days of storage. Thiobarbituric acid reactive substances (TBARS) value was not affected by PEF field strength (1.2 or 2.3 kV/cm) applied on *M. Longissimus thoracis et lumborum* pork muscles (McDonnell et al., 2014). Moreover, oxidative stability of beef (*M. Longissimus lumborum*) treated with field strengths from 0.58 to 0.73 kV/cm did not change [2]. This could be due to the low field strength applied in previous studies. Both freeze thaw and PEF processing may increase TBARS values, as parameters such as chilling and freezing as a pre- and post- treatment, and storage conditions, all have the potential to increase the lipid oxidation of PEF treated meat. A significant increase in lipid oxidation has been described with PEF treatment of frozen-thawed beef [3,5].

PEF processing can influence the volatile composition of meat. Faridnia, Ma, Bremer, Burritt, Hamid and Oey [3] reported that PEF processing of frozen thawed beef samples affected the volatile profile by increasing the protein and lipid degradation products such as dimethyl disulphide and 2,3-octanedione. Ma, Hamid, Oey, Kantono, Faridnia, Yoo and Farouk [5] further showed that PEF processing of frozen lamb meat resulted in an increase in volatile compounds such as aldehydes, including methylbutanal, hexanal, and heptanal. Both hexanal and heptanal were products of lipid oxidation during PEF processing that corresponded to an increase in TBARS values of frozen cuts. In contrast, the chilled PEF samples showed an increase in 2-heptanone, 2-ethylfuran, hexanal, heptanal, and 2,3-octanedione. However, in that study, the correlation between sensory perception and volatile composition was not investigated. It is important to determine the volatile profile changes in meat samples subjected to PEF to further understand how flavours are developed during PEF processing.

Previous studies have demonstrated that chemical properties are influenced by PEF processing of meat. The effect of PEF treatment on amino acids and fatty acids content have been reported in beef [6], seafood [7], and lamb cuts [8]. Kantono, Hamid, Ma, Oey and Farouk [8] recently showed that PEF treatment significantly changed the fatty acid composition of meat, especially, n-6 or n-3 PUFA. In contrast, PEF treatment of all chilled cuts at 0 and 7 days of storage significantly decreased fatty acids. Moreover, PEF treatment significantly increased the amino acid content of chilled knuckle, rump, topside, and loin cuts that were stored for 7 days. PEF treatment also increased the amino acid content of all the frozen meat cuts except the rib cut that were stored for 7 days. It is therefore crucial to understand how these changes in fatty acids and amino acids resulting from PEF treatments can influence volatile profiles and sensory quality of PEF processed lamb meat.

Temporal Dominance of Sensations (TDS) has been used to understand the temporal flavour profile of meat. TDS, a temporal method was chosen over a static evaluation of sensory attributes like Quantitative Descriptive Analysis (QDA) technique in order to study the progression of dominant sensory attributes in different lamb cuts with time thus providing more realistic results about flavour changes during consumption. The breakthrough idea of TDS was to no longer score intensities, but to elicit “dominance”. As dominance is far simpler to determine than scoring intensities, the TDS task is feasible for use with consumers that have some training. Consequently, TDS can be correlated to liking, wanting and/or satiation evaluated quantitatively and dynamically during consumption of food. Recently, Kantono et al. [6] investigated the effect of PEF processing on sensory properties of beef muscles (*biceps femoris* and *semitendinosus*) using TDS. The authors demonstrated that temporal flavour changes described changes in sensory quality of meat samples very well with PEF processing and storage. The current study set out to assess the effects of PEF processing of seven different lamb meat cuts (knuckle, loin, rump, rib, shank, shoulder and topside), and effects of chilled storage (0 and 7 days) of meat on free fatty acid, amino acid, flavour volatile composition, and temporal flavour profile of cooked lamb meat. Partial least Squares Regression (PLSR) analysis was further carried out to determine how chemical changes in terms of amino acids, fatty acids, and volatile composition with PEF processing and storage influenced temporal sensory perception of cooked meat during consumption.

## 2. Materials and Methods

### 2.1. Preparation of Meat Samples

In this study, chilled and frozen samples of high-value (rib, shoulder, and loin) and low-value lamb meat cuts (shank, topside, knuckle, and rump) were used. The lamb meat was obtained from AgResearch (Hamilton) at 48 h post-mortem period. The mean weight of cold carcasses was between 140.5–150.5 kg. One half of these lamb muscles were used as chilled samples which were stored at 4 °C before PEF processing. The other half were vacuum-packed and frozen at −20 °C for approximately 2 months. Each muscle was divided into five separate blocks and were stored in the vacuum packaging of polyethylene plastic bags. Frozen samples were thawed 24 h at 4 °C before PEF processing. To summarize, from a sample population of four lambs, seven different muscle samples were separated into five different parts under different storage conditions (chilled and frozen).

### 2.2. PEF Processing

Chilled and frozen samples of different lamb cuts were processed with a lab scale PEF system (Elcrack-HPV 5, DIL Quakenbruck, Germany). PEF treatment chamber (6 cm height × 4 cm width × 6 cm length) was used to hold the samples and stainless-steel parallel electrodes with a distance gap of 4 cm. The meat was carefully cut using a special cutting mould to fit the dimensions of the PEF chamber and then placed between two electrodes within the PEF chamber.

For the present study, chilled and frozen lamb cuts were studied in two different groups; control-chilled and control-frozen-thawed (non-PEF treated sample), and PEF-chilled and PEF-frozen-thawed: (PEF treated sample). The different muscles were cut parallel to the direction of fibre, which was perpendicular to the electric current. The recorded weight of sample was approximately 62 ± 5 g. PEF processing for each lamb muscle was conducted on 6 independent replicates. After processing, samples were vacuum packed in polyethylene plastic bags and were stored at 4 °C for two different storage periods (0 and 7 days). Afterwards, the samples were stored at −20 °C for approximately 2 months prior to further analysis.

Chilled and frozen samples were treated at a specific energy of 88–109 kJ kg^−1^, frequency of 90 Hz, the electric field strength of between 1–1.4 kV cm^−1^, pulse number of 964, and pulse width of 20 μs. During PEF treatment, pulse shape (square wave bipolar) was observed online through the use of an oscilloscope (Model UT2025C, Uni-Trend Group Ltd., Hong Kong, China). The initial temperature was maintained at 4 °C, and temperature of the meat cuts before and after PEF treatments was determined using temperature loggers (Grant Squirrel SQ800, Cambridgeshire, UK).

### 2.3. Sample Preparation and Temporal Dominance of Sensations (TDS) Procedure

Prior to cooking, samples were thawed at 4 °C for 24 h. Thawed samples were cooked using the *sous vide* method by immersing the vacuum-packed samples in a water bath (Model 360, Contherm, New Zealand) at 58–59 °C for 2 h. The samples were then seared using a grill (Breville BGR200BSS Healthsmart Grill, Australia). Both bottom and top plates of the grill were heated and kept at 180 °C with equal heat distribution. A lamb piece of 25 cm^3^ was quickly seared for 10 s with the bottom plate and additional 2 min with the top plate lowered. Temperature of the samples were then left to equilibrate to 80 ± 1.5 °C prior to serving the meat to panellists.

Panellist’s recruitment and training were carried out as described by Ma, Hamid, Oey, Kantono, Faridnia, Yoo and Farouk [5]. Briefly, ten panellists (5 males and 5 females) were familiarised with TDS over three training sessions (3 h per session). At the first training session, 18 relevant sensory meat attributes were chosen by panellists. After further refinement a total of five sensory attributes (meaty, browned, juicy, livery, and oxidized) were used for TDS similar to Ma, Hamid, Oey, Kantono, Faridnia, Yoo and Farouk [5] (Supplementary Table S1). Each attribute was defined and a reference for each attribute was agreed by the panellists. Panellists then carried out a dummy trial on the meat samples (5 ± 0.5 g) and rated temporal sensory perception using the TDS method using the FIZZ acquisition software v. 2.46c (Biosystemes, Counternon, France). Panellists were asked to rate the temporal changes in sensory quality of the lamb samples from the first bite, swallowing (after 30 s), and after swallowing (up to 80 s). A compulsory break of 45 s was carried out between each sample, and filtered water and water crackers were served to cleanse their mouth. Then, 24 sessions were performed to evaluate 56 samples in triplicates (1 dummy sample and 7 samples each session). Samples were coded with random three-digit numbers, and the Williams Latin Square design was used to avoid order-effect for presenting sample to panel. About 25 cm^3^ (5 × 5 × 1 cm) sample at 60 ± 0.5 °C was placed into a 100 mL cup and served to the panellists.

The Auckland University of Technology Ethics Committee approved this sensory study (AUTEC 13/317). Written and informed consent were collected for all participants. Sensory evaluation took place at the sensory laboratory located in the university. Participants received supermarket gift card for their participation in the study. Participants who were smokers, possess any sensory impairments, eating disorders, and other health problems associated with food were excluded during the recruitment process.

### 2.4. Determination of Volatile Profile Using Headspace/HS-SPME Analysis

Frozen lamb meat samples were thawed overnight at 4 °C and minced using a coffee and spice grinder (Breville, Australia). A 10 mL flat bottom headspace vial with 0.5 ± 0.1 g of minced lamb meat sample was used for HS-SPME extraction. The vial was sealed with a PTFE/ silicone septum and crimp cap (Supelco Co., Bellefonte, PA, USA). Then, headspace vial was heated to 80 °C for 5 min. This temperature was chosen to ensure the maximum release of volatiles into the headspace in accordance with our previous research [3,5]. After 5 min reset, 10 μL of an internal standard (1, 2-dichlorobenzene in methanol, 600 ppm, Sigma Aldrich, St. Louis, Missouri, USA) was added into 125 μL insert by using a HPLC syringe.

Volatile extraction was carried out according to Kantono, Hamid, Ma, Oey and Farouk [8] method with minor modification. Briefly, 50/30 μm DVB/CAR/PDMS was used for meat volatiles extraction. The vial was maintained at 60 °C in the oven during the entire extraction procedure for 30 min. Each sample was run in triplicates. A VF-5 column (Phenomenex, Torrance, CA, USA) was installed on the Trace GC Ultra (Thermo Scientific, Waltham, MA, USA) gas chromatograph. GC parameter was set up as following condition: constant helium flow rate =1.5 mL/min, oven temperature started from 40 °C (held for 3 min) and was heated to 250 °C (increasing rate = 5 °C/min, held for 3 min). The mass spectrometer was operated in the electron impact mode with a source temperature of 200 °C, an ionizing voltage of 70 eV, and the transfer line temperature was 250 °C. The mass spectrometer scanned masses from 48 to 400 *m*/*z* at a rate of 3.50 scan/s.

Semi-quantitative analysis and tentative identification of volatiles were carried out using the NIST/EPA/NIH Mass Spectral Database (National Institute of Standards and Technology, Gaithersburg, MD, version 2.0a, 2002, USA or NIST web book (http://webbook.nist.gov/chemistry/; accessed date: 18 June 2019). Retention index (RI) was also calculated with C7 to C30 n-alkanes (1000 µg/mL in hexane from Supelco. The approximate quantities of the volatiles were expressed as the ratio of individual volatile peak areas with internal standard.

### 2.5. Fatty Acid and Amino Acid Analyses

Fatty acid analysis was carried out using the fatty acid methyl ester (FAME) method as described by Ma, Hamid, Oey, Kantono, Faridnia, Yoo and Farouk [5]. Fifty milligrams of a ground, freeze-dried sample (48 h) was measured into a 4 mL amber vial. A 10 µL aliquot of tridecanoic acid (2 g L^−1^) was added as an internal standard. Toluene (490 µL) and freshly prepared 5% methanolic HCl (750 µL) were added before filling the vial with nitrogen. After incubation in the water bath at 70 °C (2 h), vials were cooled to room temperature before 6% aqueous K_2_CO_3_ (1 mL) and toluene (500 µL) were added. After centrifugation at 1100× *g* for 5 min, the top layer was removed using a glass Pasteur pipette for FAME analysis.

Derivatized methyl esters of fatty acids were separated and quantified using a Shimadzu GC 2010 with a Flame Ionisation Detector. The column used was a 0.25 mm × 30 m × 0.25 µm film thickness, fused-silica column (ZB-WAX, Phenomenex, Torrance, CA, USA). Nitrogen was used as the carrier gas, with a split ratio of 50, a head pressure of 8.7 PSI, and 1 mL/min column flow. The injector temperature was set at 250 °C. The initial oven temperature was programmed at 140 °C, then increased to 245 °C at 5 °C/min and held at 245 °C for 15 min. Thirty-seven FAME standards (Supelco product 47885-U) obtained from Sigma-Aldrich (Sydney, Australia) were serially diluted to six concentrations (from 10 to 0.3125 g L^−1^), and the standard calibration curve constructed was used for quantitative analysis.

Amino acid analysis was carried out as described by Ma, Hamid, Oey, Kantono, Faridnia, Yoo and Farouk [5] using the EZFaastTM amino acid analysis kit (Phenomenex, Macclesfield, UK). The analysis of amino acids using GC-FID was carried out according to manufacturer’s instructions. The EZ: faast™ amino acid analysis kit (Phenomenex, Torrance, CA, USA) was used to extract and derivatize the free amino acids of all lamb samples. Sample extracts were analysed using a Shimadzu GC-2010 fitted with a flame ionization detector (Kyoto, Japan), and Zebron ZB-AAA GC column (5%-phenyl-95%-dimethylpolysiloxane phase, 30 m × 0.53 mm × 1.50 µm) (Phenomenex, Inc., USA). Nitrogen was used as the carrier gas and pressure was set to 60 Pa with a 2.3 mL/min column flow rate. The oven was started at 40 °C, increased to 110 °C at 50 °C/min, then to 320 °C at 20 °C/min, and held at 320 °C for 2 min. Norvaline (0.2 mmol/L) was used as an internal standard and calibration curves of 17 amino acids (Ala, Gly, Thr, Ser, Pro, Glu, Asp, Val, Leu, Ile, Met, Phe, Lys, His, Tyr, and Trp) were used for quantification. Samples were analysed in triplicates.

### 2.6. Data Analysis

The data were collated and subjected to both univariate and multivariate statistical analysis using the XLSTAT 2020 (Addinsoft, New York, NY, USA) software. TDS curves and TDS band plots were generated using the FIZZ Calculations software (FIZZ Calculations v2.46c, Biosystemes). Mixed-model analysis of variance (ANOVA) was carried out on volatile composition of PEF processed samples. If a significant difference was observed (α = 0.05), the Fisher’s LSD test was run to establish differences between means.

Multivariate approach using Canonical Variate Analysis (CVA) and Hotelling–Lawley trace Multivariate Analysis of Variance (MANOVA) were carried on the total duration of each dominant attribute for all samples evaluated in order to create an overall product sensory map with 90% confidence ellipses plotted for each product centroids [9]. CVA approach was utilized in this study as it maximizes the distances between products through discriminative functions while minimizing residual variability [10].

Additionally, Partial Least Square Regression (PLSR) analysis was also carried out to investigate the relationship between chemical and sensory measures. In the PLSR model, the chemical data from volatile composition, amino acids, and fatty acids were treated as the independent variables (X matrix), with the sensory data obtained from PEF treatment of different lamb cuts used as the dependent variables (Y matrix). In addition, Variable Importance in Projection (VIP) was also calculated with limits on 0.8 and 1, corresponding to 90% and 95% confidence interval.

## 3. Results and Discussion

### 3.1. Sensory Evaluation

#### 3.1.1. Panel dominance curves

**Figure 1:**
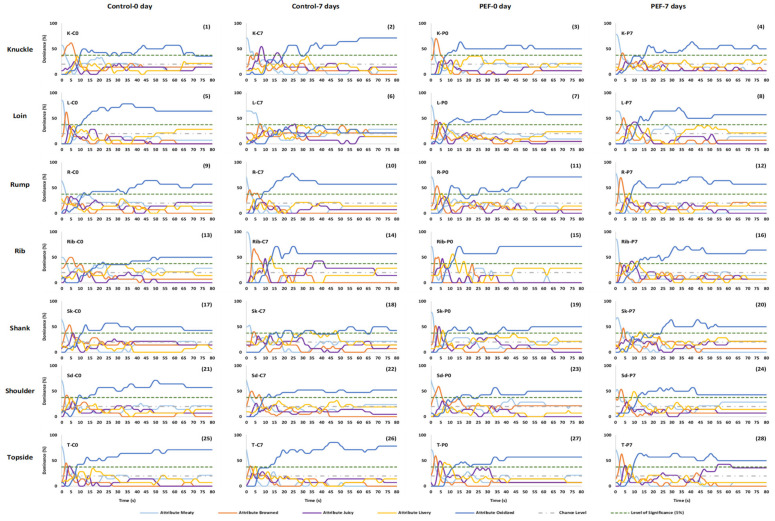
TDS curves of non-PEF and PEF treated cooked chilled lamb meat cuts (C: control; P: PEF; 0 and 7: number of storage days).

**Figure 2:**
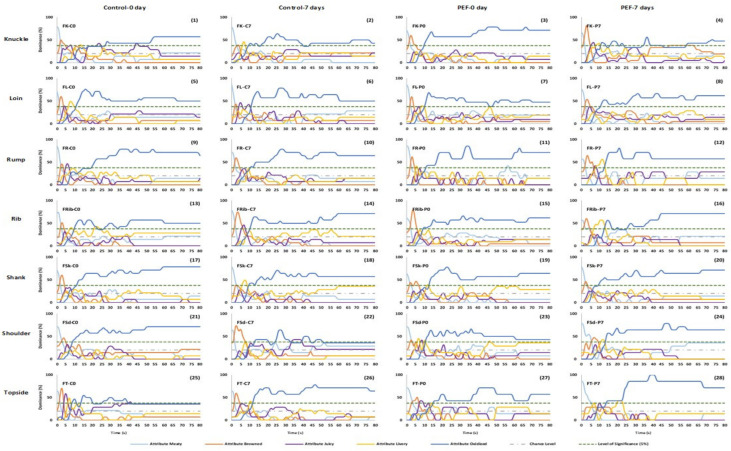
TDS curves of non-PEF and PEF treated cooked frozen-thawed lamb meat cuts (C: control; P: PEF; 0 and 7: number of storage days).

The significance level of this study was 0.37 (37% dominance rate) in this study. TDS curves successfully discriminated the effect of aging and different cuts of dominant sensory attributes of the samples. All samples have found that dominance of meaty, browned, juicy and livery attributes during the mastication period, and then oxidized thereafter (Figure 1 and Figure 2). Meaty was the first dominant sensation in all samples with the dominance rates starting at over 50% dominance rate and then decreasing to chance level within 5 s. Dominance of the browned attribute reached significance in the first 3--5 s. Starting from 10 s, oxidized became the dominant attribute above significance level until the end of mastication.

Figure 1 shows TDS curves of different PEF-treated chilled lamb cuts. In the knuckle cut (1–4), meaty started at around 70% dominance rate (except for K-C0 (1)), and then decreased to below chance level at 5 s. Only K-C0 (1) and K-P0 (3) samples were dominant in browned attribute for a short time between 2–7 s and achieved to 60 % and 65 % dominance rate at 5 s, respectively. Juicy was only significant in K-C7 (2) sample. Oxidized became significant in almost all samples from 10 s onwards except for K-C7 (2) that was dominant in oxidized attribute only after 20 s. Interestingly, juicy was dominant in K-C7 (2) sample between 7–10 s and 17–19 s. In loin cuts (5–8), almost all samples were dominant in browned attribute for a short time between 2–5 s except for L-C7 (6) sample. Almost all loin cuts were dominant in oxidized attribute after 10 s thereafter except for L-C7 (6) sample. The browned attribute was dominant in almost all rump cuts except for R-C0 (9), with a maximum 70% dominance rate at 3 s in R-P7 (12) only. Oxidized became dominant in rump cuts after 10 s onwards, except for R-C0 (9) where oxidation became dominant only after 16 s. In rib cuts, meaty was dominant above significance level in the first 3 s. The browned attribute then became dominant from 5 until 10 s, except for Rib-P7 (16) that did not reach significance level for this attribute. Juicy was not present in Rib-C0 (13) sample but was dominant in Rib-C7 (14), Rib-P0 (15) and Rib-P7 (16) samples between 9 and 11 s. Similar to juicy, livery was dominant in Rib-C7 (14) and Rib-P0 (15) above significance levels. Oxidized then became dominant above significance levels in almost all rib samples except for Rib-C0 (13) from 12 s onwards. Shank cuts were dominant in browned attribute between 2–5 s above significance level except for Sk-C7 (18). Oxidized was significant in all shank cuts that became dominant from 10 s, except for Sk-P7 (20) when oxidized became dominant after 25 s. In general, dominance of the oxidized attribute was generally less than 50% for all samples. As for shoulder cuts, meaty started at around 70% dominance rate for the first 3 s except for Sd-P7 (24). Browned became dominant from 3–8 s above significance level for almost all shoulder samples except Sd-C0. Similar to shank cuts, dominance of the oxidized attribute was generally less than 50% for almost all shoulder samples except for Sd-C0 (21). In topside cuts, browned was dominant in T-P0 (27) and T-P7 (28) samples with over 55% dominance rate between 3 and 8 s. Juicy and livery were dominant in T-P0 (27) and T-P7 (28) samples but less than 50%. Oxidized was significant in all topside samples between 8 and 15 s. Dominance of T-P0 (27) and T-P7 (28) were just above 50% or less compared to control samples.

Figure 2 shows the TDS curves of sensory attributes of different PEF-treated frozen-thawed lamb cuts. In knuckle cut (1–4), meaty was the most dominant at the start of mastication in FK-C0 (1), FK-C7 (2) with 80% dominance rate and FK-P0 (3), FK-P7 (4) with 70% dominance rate, respectively. Browned was dominant in almost all knuckle samples (except FK-C7 (2)) between 2 and 5 s and FK-P7 (4) had higher (80%) dominance rate compared to others. The livery was only dominant in FK-C7 (2) and FK-P7 (4) samples, between 6 and 8 s, and 8 and 10 s, respectively. Oxidized was dominant in FK-C7 (2) and FK-P0 (3) from 10 s and became dominant after 16 s in FK-C0 (1). Oxidized also fluctuated around the significant level in FK-P7 (4). In loin cut (5–8) samples, meaty was dominant in all samples and then dramatically dropped to above chance level at 2 s. Livery was only dominant at 9 s in FL-P0 (7) sample. Juicy in FL-C7 (6) sample was dominant from 4 to 10 s and then dropped to above chance level. In rump cuts (9–12), browned attribute in FR-C0 (9), FR-C7 (10), and FR-P0 (11) samples was dominant from 3 to 5 s. The browned attribute in FR-P7 (12) (3.0–8.0 s) had longer duration compared to other rump cuts. Juicy was only dominant in FR-C0 (9) and FR-P7 (12) samples between 5 and 8 s. In addition, livery was only dominant in FR-P7 (12) between 5 and 12 s. For all rib cut samples (13–16), meaty was the most dominant at the start of mastication and then decreased to below chance level in the first 10 s. FRib-C7 (14) sample was only significantly dominant in juicy attribute from 5 to 10 s. Livery was only dominant in FRib-P7 (16) sample from 8 to 15 s. The oxidized attribute became dominant at 10 s in FRib-C0 (13) and FRib-C7 (14) samples, while in FRib-P0 (15) and FRib-P7 (16) samples oxidized became dominant at 12 s. For shank cut (17–20) samples, browned attribute in FSk-C0 (17) and FSk-P7 (20) samples were dominant from 2 to 4 s. It can be seen that the browned attribute in FSk-P0 (12) (2.0–7.0 s) had a longer duration compared to FSk-C0 (17) and FSk-P7 (20) samples. Livery was only dominant in FSk-C7 (18) sample from 5 to 8 s. In the shoulder cut samples (21–24), meaty was initially dominant until 3 s. However, for FSd-P7 (24) sample, meaty was additionally 2 s longer (0–5.4 s). Browned attribute in FSd-C0 (21) and FSd-C7 (22) samples was dominant from 3 to 5 s. It can be seen that the browned attribute FSd-C0 (21) and FSd-P0 (23) (1.7–3.0 s) lasted for a shorter duration compared to FSd-C7 (22) (0.8–7.5 s) and FSd-P7 (24) (5.2–9.2 s) samples. Livery was only dominant for FSd-P0 (23) and FSd-P7 (24) samples, from 6 to 7 s and 10–12 s, respectively. Oxidized was dominant in FSd-C0 (21) and FSd-C7 (22) samples that increased at 9 s and remained stable thereafter with small fluctuations until the end of mastication. However, FSd-P0 (23) and FSd-P7 (24) oxidized increased in dominance after 12 s, and in FSd-C7 (22) sample dropped to just below significance level after the 50 s. In the topside cut (25–28), meaty attribute in FT-C0 (25), FT-C7 (26), and FT-P0 (27) samples was dominant from 0 to 4 s. It can be seen that the browned attribute in FT-P7 (28) (0–6.0 s) had longer duration compared to other topside samples. Browned attribute was significant in FT-C0 (25), FT-C7 (26), and FT-P0 (27) between 2 and 5 s. The juicy attribute was only dominant in FT-C0 (25) and FT-P0 (27) between 4–7 s and 6–9 s. Oxidized attribute in FT-C0 (25) was dominant between 6 and 40 s, which was at least 35 s shorter in duration compared to FT-C7 (26) (10.0–80.0 s), FT-P0 (27) (15.0–80.0 s), and FT-P7 (28) (12.0–80.0 s).

#### 3.1.2. Canonical Variate Analysis

**Figure 3:**
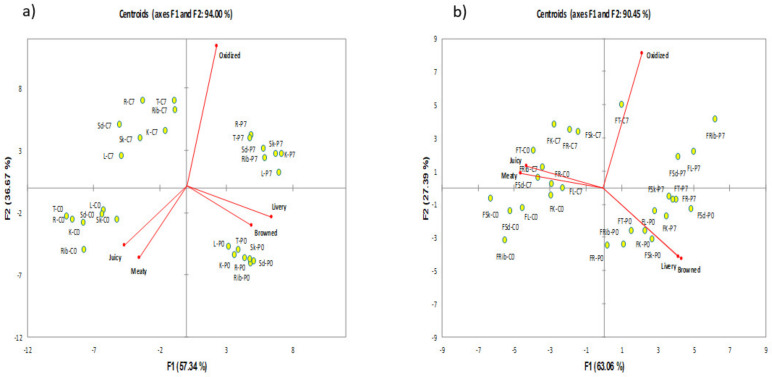
Canonical Variate Analysis Biplot of dominance durations of sensations of PEF and non-PEF treated cooked chilled (**a**) and frozen-thawed (**b**) lamb meat of different cuts. Hotelling–Lawley trace MANOVA test showed significant product differences ((**a**): F_(135,192)_ = 29.655; *p* < 0.001; (**b**): F_(135,192)_ = 12.043; *p* < 0.001) based on sensory attributes. Sd = Shoulder; Rib = Rib; L = Loin; K = Knuckle; R = Rump; S = Shank; T = Topside; C: control; P: PEF; 0 = 0 days storage; 7 = 7 days storage/post-processing storage.

TDS curves only provided dominance rate, which can be translated to % of panel agreement and does not measure intensity. It was also shown on Figure 1 and Figure 2 that oxidized was the most dominant attribute throughout consumption, which unfortunately did not discriminate samples. Hence, a CVA was applied in the TDS duration data in order to provide a clearer interpretation of the TDS results. In this study, both chilled (Figure 3a) and frozen-thawed (Figure 3b) samples were discriminated by CVA with a high variance for sensory data of 94.00% and 90.45%, respectively. 

For the chilled meat samples (Figure 3a), Hotelling–Lawley MANOVA (F_(135,192)_ = 29.655; *p* < 0.01) showed significant differences between the samples in terms of the temporal flavour attributes measured by TDS. F1 explained 57.34% of the variance, separating the meat samples in terms of PEF processing, where negative scores of the CVA corresponded to control non-PEF samples, and positive scores corresponded to PEF treated samples. F2 (36.67%) further distinguished between the 0- and 7-days storage meat samples. Positive scores of F2 corresponded to 7 days storage samples, while negative scores corresponded to 0-days storage samples. 7 days samples were correlated with oxidized flavour. In contrast, control samples (non-PEF) at 0 days were positively correlated with meaty and juicy. PEF treated samples stored for 0 days were correlated with browned and livery attributes.

In terms of frozen-thawed meat samples (Figure 3b), F1 explained 63.06% of the variance. Meat samples were separated along F1 in terms of PEF processing treatment, where negative scores of the CVA corresponded to non-PEF control samples (except FT-C7), and positive scores corresponded to PEF treated samples. F2 (27.39%) further discriminated almost all lamb samples between the 0- and 7-days storage. Positive scores of F2 were associated with 7 days storage samples (except FT-C0 and FR-C0), while negative scores were associated with 0 days samples (except FR-P7, FSk-P7, FT-P7, FSd-P0, and FK-P7). Samples treated with PEF were correlated with oxidized flavour, especially FRib-P7. In contrast, control samples (non-PEF) were positively correlated with meaty and juicy, especially at 0-days storage. PEF treated 0-days samples were correlated with browned and livery attributes.

### 3.2. Volatile Analysis

In this study, 24 volatiles were detected by GC–MS, which included 1 alcohol, 3 ketones, 7 aldehydes, 3 furans and 10 nitrogen- and sulphur-compounds (Supplementary Tables S2 and S3).

#### 3.2.1. Effects of Storage, PEF Processing, and Cuts on Volatile Composition

##### PEF-Treated Chilled Samples

**Figure 4:**
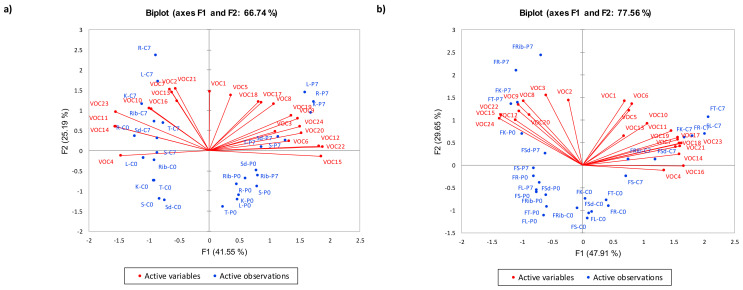
Principal Component Analysis Biplot of volatile profiles of PEF and non-PEF treated cooked chilled with 66.74% variance explained (**a**) and frozen-thawed with 77.56% variance explained (**b**) lamb meat of different cuts. Sd = Shoulder; Rib = Rib; L = Loin; K = Knuckle; R = Rump; S = Shank; T = Topside; C: control; P: PEF; 0 = 0 days storage; 7 = 7 days storage/post-processing storage. VOC codes for Figure 4a correspond to Supplementary Table S2 for chilled samples, and codes in Figure 4b correspond to Supplementary Table S3 for frozen samples.

Principal Components Analysis (PCA) provides an overview of how storage, PEF processing, and different meat cuts interacted in a 2-dimensional product space. Figure 4a shows that different meat cuts did not have any significant effect on volatile composition. Interestingly, factor 1 (F1) differentiated control samples from PEF processed samples stored for 7 days. In addition, factor (F2) differentiated the samples in terms of effects of storage.

Figure 4a explained 66.74% variance and showed that PEF-treated samples were separated along F1 from control samples. Positive scores along F1 were correlated with PEF processed samples stored for 7 days. Chilled samples that were subjected to both PEF processing and 7 days storage were associated with 2-heptanone (3), 3-methyl-butanal (6), hexanal (8), heptanal (9), 2-ethylfuran (12), pyridine (15), methyl-pyrazine (17), 2-ethyl-3-methyl-pyrazine (18), 3-ethyl-2,5-dimethyl-pyrazine (19), 3,5-diethyl-2-methyl-pyrazine (20), dimethyl disulphide (22), and dimethyl trisulphide (24). Pyrazines are generally associated with non-enzymatic browning, which generally possesses roasted, nut-like notes [11]. Aldehydes like 3-methyl-butanal (6), hexanal (8), and heptanal (9) were another major class of volatiles that was associated with both PEF processing and 7-days storage. The presence of aldehydes has been associated with lipid oxidation in meat. For example, hexanal occur in meat during flavour formation and lipid oxidation and heptanal arises from the oxidation of oleic acid [12,13]. The volatile, 3-methylbutanal (6) described as being malty and fatty has been reported to be an important volatile compound in cooked beef [12] and goat [14] that can contribute to roasted beef flavour [12]. In addition, Ruiz et al. [15] reported that 3-methylbutanal was found in high concentrations in longer aged hams. Sulphur volatile compounds are derived from sulphur containing amino acid degradation [16]. The potent sulphur-containing compounds can be formed from the reaction between cysteine and ribose when meat is cooked. Dimethyl sulphide, dimethyl trisulphide, and dimethyl disulphide have been detected in beef [17].

In contrast, negative scores along F1 were correlated with control samples that were stored for 7 days. Control samples that were subjected to 7 days storage were shown to be associated with 2,3-octanedione (2), 2-nonanone (4), 2-methyl-butanal (7), benzaldehyde (10), nonanal (11), 2-vinylfuran (13), 2-pentylfuran (14), pyrrole (16), thiophene (21), and 2-furanmethanethiol (23). Similarly, 2,3-octanedione and 2-pentyl-furan have been reported in chilled aged beef muscles for 7 or 14 days [18]. These volatiles are often associated with lipid oxidation and are affected by enhancement and aging in the various muscles. Ma, Hamid, Oey, Kantono, Faridnia, Yoo and Farouk [5] also reported that the relative concentration of the lipid oxidation product (2-pentylfuran) increased in non-PEF treated (control) shoulder and rib cuts of lamb meat with increased storage time.

PEF processed and control chilled samples that were stored at 7 days storage had positive scores along F2 that were associated with 1-hexanol (1) and 2-methyl propanal (5), 2,3-octanedione (2) and thiophene (21). The alcohol, 1-hexanol (1), derived from hexanal reduction, was present in the shoulder cut sample at significantly lower levels as compared to other cuts. In contrast, chilled rib cut had a lower concentration of 2-methyl propanal as compared to other cuts. Generally, thiophenes, sulphur containing compounds, and furans can contribute to meat-like aromas that have exceptionally low odour threshold values [16]. Overall, storage for 7 days led to the production of more volatiles compared day 0 samples. Increased volatile production with increased storage period can be a result of lipid oxidation as the ability of unsaturated fatty acids to oxidize plays an important role in the flavour development during cooking [19]. Kantono, Hamid, Oey, Wang, Xu, Ma, Faridnia and Farouk [6] also reported that TBARS values (between 0.06 and 0.12 mg MDA/kg) of fresh beef samples increased (although below the critical level that contributes to rancidity) with increased lipid oxidation after 7 days of storage.

##### PEF-Treated Frozen Samples

Similar to chilled samples, F1 differentiated control frozen samples from PEF processed frozen samples when stored for 7 days. In addition, F2 differentiated the frozen samples in terms of effects of storage. Figure 4b explained 77.56% variance and showed that samples subjected to PEF treatment were separated along F1 from control samples. Negative scores along F1 were correlated with PEF processed samples stored for 7 days. Most frozen lamb cuts that were subjected to both PEF and 7 days storage were closely associated with 2,3-octanedione (2), 2-heptanone (3), hexanal (8), heptanal (9), 2-ethylfuran (12), pyridine (15), 3,5-diethyl-2-methyl-pyrazine (20), dimethyl disulphide (22), and dimethyl trisulphide (24). In contrast to chilled samples, frozen-thawed PEF treated samples stored for 7 days were not mainly dominated by pyrazines. The volatile compound, 2,3-octanedione, can be derived from the heating and breaking down of linoleic acid [20]. Stetzer, Tucker, McKeith and Brewer [18] reported that 2,3-octanedione had an oxidized fat and warmed-over flavour, which is derived from lipid oxidation. This result can be explained by higher lipid oxidation in frozen-thawed cuts. Since, sulphur volatile compounds are derived from sulphur containing amino acid degradation [16], freezing condition and longer storage may have accelerated protein degradation in meat. The presence of more sulphur volatile compounds in aged meat may also be due to the increased concentration of cysteine during storage [21]. Frozen samples that were subjected to PEF treatment and 7-day storage period were also associated with aldehydes like hexanal (8) and heptanal (9). In terms of cuts, significantly higher level of hexanal and heptanal was present in frozen-thawed rump cut and shank cut, respectively.

Positive scores along F1 were correlated with control frozen samples that were stored for 7 days. Control samples that were subjected to 7 days storage were shown to be associated with 2-nonanone (4), 2-methyl-butanal (7), nonanal (11), 2-pentylfuran (14), pyrrole (16), methyl-pyrazine (17), 2-ethyl-3-methyl-pyrazine (18), 3-ethyl-2,5-dimethyl-pyrazine (19), thiophene (21), and 2-furanmethanethiol (23). In this study, the frozen-thawed topside sample had a significantly higher level of methyl-pyrazine (17), 2-ethyl-3-methyl-pyrazine (18), and 3,5-diethyl-2-methyl-pyrazine (20) than other cuts (except knuckle and rib). Moreover, large amounts of 2-furanmethanethiol (23) were also present in the meat sample, with the frozen-thawed loin and rump cuts having significantly higher levels of this compound compared to shoulder and shank cuts. The volatile, 2-furanmethanethiol (furfuryl mercaptan), was reported to be formed from the breakdown of ribose via the Maillard reaction [22]. It possesses roast, nutty, burnt, and meaty notes, which contribute to cooked goat and chicken aroma [14,16].

Most PEF-treated and control frozen meat cuts that were stored at 7 days storage had positive scores along F2 and were associated with 2,3-octanedione (2) and 2-heptanone (3). A ketone, 2-heptanone (3) was present in significantly higher levels in frozen-thawed rump cut. In contrast, 2,3-octanedione (2) was present in significantly higher levels in and frozen-thawed shank cut. Overall, storage period of 7 days led to the production of more volatiles as compared to 0 day samples. Similar to chilled samples, this can be attributed to the increase in the lipid oxidation with increased storage. Kantono et al. [8] also found that increased storage period led to a significant increase in the lipid oxidation that corresponded to increased TBARS value in frozen lamb cuts.

### 3.3. Partial Least Squares Regression Analysis to Determine the Relationship between Sensory and Chemical Measures

PLSR analysis is widely applied to elucidate relationships between instrumental and sensory data in a multivariate way. In order to clearly understand the relationship between sensory characteristics and volatile composition, as well as fatty acid amino acid composition, PLSR analysis was performed. Fatty acids and amino acids results from a recently published study by Kantono, Hamid, Ma, Oey and Farouk [8] on the effects of PEF processing on chilled and frozen thawed lamb cuts were used to further understand how these chemical measures were correlated to sensory flavour perception.

#### 3.3.1. Variable Importance in Projection (VIP)

For assessment of relative variable importance in each PLSR model, the information of each variable (representing a signal measurement at a specific time point) was assessed by its VIP [23]. In this study, VIP scores for each variable in the partial least-squares regression (PLSR) models arising from analysis of fatty acids, amino acids, and volatile composition were examined in relation to sensory attributes. VIP scores for each explanatory variable were examined to determine their relative importance towards sensory perception. This allows a quick identification of explanatory variables that contribute the most to sensory perception.

#### 3.3.2. Chilled Samples

**Figure 5:**
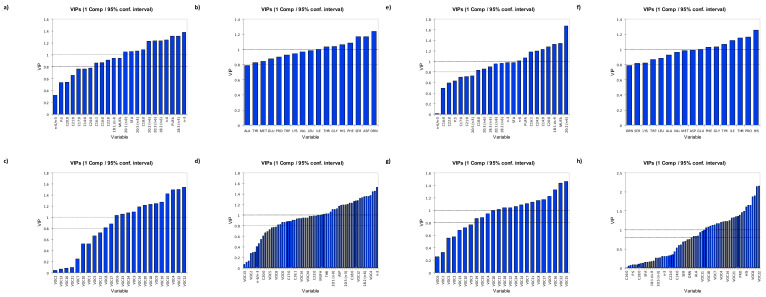
(**a**–**d**). Variable importance in the projection (VIP) for each variable in the partial-least-squares regression (PLSR) models arising from analysis of PEF treated fresh lamb samples in terms of (**a**) fatty acids, (**b**) amino acids, (**c**) volatile composition; (**d**) fatty acids, amino acids, and volatile composition were examined in relation to sensory attributes. (**e**–**h**) VIP for each variable in the PLSR models arising from analysis of PEF treated frozen lamb samples in terms of (**a**) fatty acids, (**b**) amino acids, (**c**) volatile composition; (**d**) fatty acids, amino acids, and volatile composition were examined in relation to sensory attributes. VOC codes for Figure 5a–d correspond to Supplementary Table S2 for chilled samples, and codes in Figure 5e–h correspond to Supplementary Table S3 for frozen samples.

For PEF treated chilled samples, C20:2 (n-6), MUFA, C18:1 cis-9, C20:0, C14:0, C24:0, C16:1, PUFA, and n-6 fatty acids, had VIP scores of >1 indicating that they are the most important variables in relation to sensory perception (Figure 5a; Supplementary Tables S4 and S5). Fatty acid profile of meat can influence meat flavour [24]. Similar to findings in the present study, MUFA, especially C18:1 cis-9, was positively correlated with flavour intensity and overall palatability in beef [3]. This positive correlation was attributed to C18:1 cis-9 that gives a juicier mouthfeel to meat [25].

Interestingly, all the amino acids were important variables contributing to sensory flavour perception. Histidine, proline, threonine, isoleucine, tyrosine, glycine, phenylalanine, and glutamate were the highest contributors to overall flavour perception of chilled lamb samples (Figure 5b). A large number of small peptides and free amino acids generated in dry-cured meats can influence the development of taste, flavour precursors, and aroma attributes [21]. Similar to the present study, Koutsidis, Elmore, Oruna-Concha, Campo, Wood and Mottram [21] reported that free amino acids like threonine, phenylalanine, isoleucine contributed to brown/roasted flavour of beef, which were desirable to consumers. Moreover, free amino acids like tyrosine, phenylalanine, and threonine contributed to the tenderisation and flavour enhancement in lamb longissimus dorsi muscles treated with chilled ageing conditioning at 4 °C on the 8th day of storage [26].

Nearly half of the flavour volatiles determined in this study contributed to the overall flavour perception of chilled samples of lamb. Pyridine, dimethyl disulphide, pyrrole, heptanal, methyl-pyrazine, hexanal, thiophene, 2-methylbutanal, 2-pentylfuran, dimethyl trisulphide, 2-ethylfuran, 2-ethyl-3-methyl pyrazine, and nonanal were the highest contributors of chilled lamb samples flavour (VIP > 1) (Figure 5c). Similarly, Machiels [12] identified dimethyl sulphide, 2-butanone, 2-and 3-methylbutanal, dimethyl trisulphide, nonanal, and ethyl acetate as key flavour components in cooked Irish Angus beef. Among the aldehydes, hexanal, heptanal, and nonanal were also identified as important flavour components in cooked beef by Machiels [12]. Heterocyclic and non-heterocyclic compounds containing sulphur and nitrogen are important flavour compounds produced during the Maillard reaction that contribute to meaty, savoury, roasty, and boiled flavour characteristics [14,16]. Dimethyl and trimethyl sulphide found in this study possessed characteristic meat-like taste as confirmed by GC-O analysis of lamb aroma [27]. Pyrazines that result from the Maillard reaction between free amino acids and reducing sugars are important flavour components that confer characteristic “grilled meat” roasted-like flavour [11]. According to Gkarane et al. [28], a positive correlation was established between pyrazines (2-ethyl-3,5-dimethyl pyrazine, 2,5-dimethyl pyrazine, and 2-ethyl-3,6-dimethyl pyrazine) and sensory attributes like roast meat flavour of grilled lamb.

When taken together, the most important components that contributed to the overall sensory flavour perception of PEF treated chilled lamb samples were the flavour volatiles (dimethyl disulphide, pyridine, heptanal, hexanal, 2-ethylfuran, pyrrole, dimethyl trisulphide, 2-heptanone, 3,5-diethyl-2-methyl pyrazine, 2,3-octanedione, thiophene, methyl pyrazine, 2-pentylfuran, 2-nonanone, 2-methylbutanal, and 2-ethyl-3-methyl pyrazine) and amino acids (histidine, proline, glycine, glutamic acid, tryptophan, isoleucine, tyrosine, threonine, methionine, phenylalanine, and aspartic acid) (Figure 5d). In terms of PEF treated chilled lamb samples, amino acids predicted the sensory quality of meat better than fatty acids, because of their role in the Maillard reaction and Strecker degradation. These reactions are important pathways that contribute to the formation of volatile compounds in cooked meat [22]. These volatile compounds, in turn, contribute to meaty, savoury, roast, and boiled flavours [16]. For instance, sulphur compounds produced from reaction with ribose and cysteine are specifically critical for a characteristic meat aroma [16].

#### 3.3.3. Frozen Samples

In terms of PEF treated frozen samples, n-3, C18:3 (n-3), PUFA, n-6, C18:2 (n-6), C20:3 (n-6), C20:2 (n-6), C18:0, C20:5 (n-3), SFA, and C20:1 (n-9) fatty acids had VIP scores of > 1 indicating that they are the most important variables in relation to sensory perception (Figure 5e; Supplementary Tables S6 and S7). Costa et al. [29] attributed the increase in flavour of frozen *longissimus lumborum* lamb muscles to the increase in PUFA content, especially n-3 PUFA content. This is because PUFAs are generally more prone to oxidative degeneration, which results in the production of volatile flavour compounds in meat. Moreover, Nute et al. [30] reported that n-3 PUFA, mainly C18:3 (n-3) (a-linoleic acid) showed the strongest positive correlation with lamb flavour. Fisher et al. [31] also found that when concentration of C18:3 (n-3) fatty acid increased, the score for lamb flavour intensity and flavour liking significantly increased.

Among the amino acids, ornithine, aspartic acid, serine, phenylalanine, histidine, glycine, threonine, and isoleucine amino acids were the important contributors (VIP > 1) to the sensory perception of PEF treated frozen lamb samples (Figure 5f). The presence of free amino acids like phenylalanine, isoleucine, threonine, and serine in the frozen samples of lamb can contribute to brown/roasted attribute, which is desired by consumers [21]. Free amino acids like isoleucine and phenylalanine contributed to the flavour of frozen beef loins [32]. Moreover, glycine, aspartic acid, alanine, phenylalanine, tyrosine, and glutamic acid have been collectively called as flavour amino acid (FAA) because of their contribution to the flavour and freshness in imported and local chicken breast, beef meat, and Nile tilapia fish frozen at −7 °C for 1 month and −18 °C for 3 and 6 months [33].

Flavour volatiles that contributed to the overall sensory perception of PEF treated frozen lamb samples included 2-ethylfuran, dimethyl disulphide, 2-nonanone, pyridine, 2,5-dimethyl-3-ethyl pyrazine, heptanal, 2-ethyl-3-methyl pyrazine, 3,5-diethyl-2-methyl pyrazine, 2-pentylfuran, 2-heptanone, dimethyl trisulphide, and 2-furanmethanethiol, 3-methylbutanal (VIP > 1) (Figure 5g). Sulphur compounds like dimethyl sulphide and dimethyl trisulphide significantly contributed to the aroma of pork (frozen at −80 °C), and this was attributed to their high reactivity as they can either react with each other or with other compounds, leading to the production of more complex aroma compounds [34]. In addition, pyrazines that generally result from the Maillard reaction and Strecker degradation also produced important flavour volatiles for PEF-treated frozen samples. Raes et al. [35] reported that Irish beef frozen at −20 °C had the highest concentration of pyrazines, which contributed to roasted beef flavour of Irish beef. In contrast to chilled samples, ketones were one of the highest contributors to flavour of PEF treated frozen lamb. Caporaso et al. [36] identified 2-nonanone, a methyl ketone, as one of the most important compounds in defining the flavour of lamb/mutton.

When considered together, the most important components that contributed to the overall sensory flavour perception of frozen samples were fatty acids (n-3, C18:3 (n-3), PUFA, n-6, C20:3 (n-6), C18:2 (n-6), C20:2 (n-6), C18:0, C20:5 (n-3), SFA, C20:1 (n-9), MUFA, C18:1 cis-9), flavour volatiles (2-nonanone, 2-ethyl-3-methyl pyrazine, 2-ethylfuran, dimethyl disulphide, 2,5-dimethyl-3-ethyl pyrazine, pyridine, heptanal, 3,5-diethyl-2-methyl pyrazine), and amino acids (ornithine, serine, aspartic acid, phenylalanine, histidine, threonine, isoleucine and glycine) (Figure 5h). Fatty acids predicted the sensory perception of PEF treated frozen samples better compared to chilled samples. This is because lipid oxidation is the principal reaction that takes place in frozen raw and cooked meats [37] which further leads to the development of volatile compounds that directly impact meat flavour. For instance, methyl ketones found in this study are generally produced by thermal β-oxidation of the saturated fatty acid followed by decarboxylation [37].

### 3.4. PLSR Correlations between Chemical Composition and Sensory Perception

#### 3.4.1. Chilled Samples

**Figure 6:**
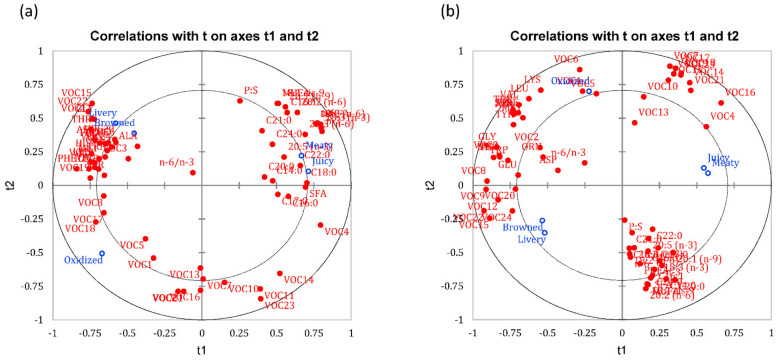
(**a**) Correlation biplots of Partial Least Square Regressions (PLSR) models for PEF treated chilled samples representing fatty acids, amino acids, and volatile composition in relation to sensory attributes. (**b**) Correlation biplots of PLSR models for PEF treated frozen samples representing fatty acids, amino acids, and volatile composition in relation to sensory attributes. VOC codes for Figure 6a correspond to Supplementary Table S2 for chilled samples, and codes in Figure 6b correspond to Supplementary Table S3 for frozen samples. Exploratory variables with VIP less than 1 are omitted for clarity.

The flavour perception of juicy and meaty were closely associated with C18:0, SFA, 20:5 (n-3), and n-3 that had positive loadings along Dimension 1. Interestingly, oxidised was negatively correlated with all fatty acids measured in this study (Figure 6a). Similar to Yancey et al. [38], medium and long-chain unsaturated fatty acids yielded oxidised and liver-like off flavours. Moreover, Hwang and Joo [39] indicated that the majority of SFAs were positively correlated with flavour intensity, tenderness, and juiciness in beef. However, a negative association were observed between flavour intensity, tenderness, and juiciness, and PUFA in Hanwoo steers [40].

The majority of amino acids (isoleucine, phenylalanine, histidine, threonine, glycine, ornithine, aspartic acid, serine) found in this study were correlated to oxidised, browned, and livery flavour that had negative loadings along Dimension 1 (Figure 6a). Similarly, Koutsidis, Elmore, Oruna-Concha, Campo, Wood and Mottram [21] reported that amino acids such as serine, threonine, valine, leucine, isoleucine, and phenylalanine contributed to brown or roasted attribute. In another study, when three different lamb cuts were subjected to high-pressure treatment at 400 MPa and above, attributes such as livery, browned, and oxidised were mainly associated with the presence of amino acids [25].

The correlation biplot (Figure 6a) of PEF treated chilled lamb samples showed that livery and browned flavour were closely associated with 3-methyl-butanal, 2-ethylfuran, pyridine, and 3,5-diethyl-2-methyl- pyrazine that had high negative loadings along Dimension 1. The formation of 3-methyl-butanal flavour from the Strecker (Maillard) reaction is commonly related to browned flavour [41]. The 2-ethylfuran volatile compound mainly resulted from the degradation of lipids, and they are sometimes regarded as markers of lipid oxidation [42]. Mussinan and Walradt [43] found that most of the compounds associated with livery flavour in pork liver were pyrazines. The oxidised flavour was closely associated with 2-ethyl-3-methyl pyrazine that had high negative loadings along Dimension 2. The volatile, 2-ethyl-3-methyl pyrazine has been associated with earthy, oily, and nutty flavour in peanuts [44] and potatoes [45].

A correlation biplot showing how fatty acids, amino acids, and volatile composition were correlated to sensory attributes is shown in Figure 6a. Meaty and juicy attributes that had high loadings along Dimension 1 were separated from livery and browned that had high negative loadings. Meaty and juicy attributes were associated with the presence of fatty acids. Meaty and juicy are two positive attributes of meat flavour that are correlated with the presence of fatty acids because fats can impact flavour in two ways; oxidation of fatty acids produces carbonyl compounds, which are strong flavour contributors, and fatty acids that can also act as a storage repository for odoriferous compounds, which are released on heating [46]. Fatty acids primarily influence flavour and juiciness [47]. Perceived juiciness is impacted through lubrication by fatty acids and stimulation of saliva during mastication. Volatile flavour compounds have been demonstrated to increase as overall fatty acid content increases. This may be due to the retention of fat-soluble volatile compounds leading up to consumption. Moreover, Smith and Carpenter [48] specified that while the general meaty flavour is non-lipid in origin, some amount of fat is crucial to make beef flavour full and rich. Livery and browned were mostly associated with the presence of amino acids and some volatiles especially pyridine, dimethyl disulphide, 2-ethyl furan, and 3,5-diethyl-2-methyl pyrazine. Livery notes can be contributed by dimethyl disulphide. Insausti et al. [49] demonstrated that levels of the sulphur-compounds in cooked beef were closely associated with blood and liver notes and unpleasant flavours. The association between some volatiles and amino acids with livery and browned flavour can be attributed to the Maillard reaction or formation of Amadori compounds. Amino acids are one of the key ingredients in Maillard browning which reacts with sugar. Additionally, amino acids could also undergo oxidative browning to form Amadori compounds, which contributes to browned flavour.

#### 3.4.2. Frozen Samples

The correlation biplot (Figure 6b) of PEF treated frozen lamb samples showed that juicy and meaty were associated with C14:0, C16:1, C20:0, and C24:0 that had high positive loadings along Dimension 2. Similar to chilled samples, the majority of saturated fatty acids were positively correlated with positive flavours like juicy and meaty. In accordance with the present results, saturated fatty acids such as C14:0 were associated with positive sensory perception (e.g., juiciness) of Korean Hanwoo beef frozen at −20 °C [50]. Livery and browned were associated with PUFA, MUFA, n-6, 18:1 cis-9, and 20:2 (n-6) on the negative loadings of Dimension 2. High concentrations of unsaturated fatty acids has been reported with formation of undesirable off-odours and flavours in meat due to lipid oxidation [51]. The presence of the aforementioned unsaturated fatty acids was also reported to be associated bloody, metallic, and livery flavours in beef loin samples [52].

The correlation biplot (Figure 6b) of PEF treated frozen lamb samples showed that amino acid measurements for frozen samples were correlated with oxidised, browned, and livery flavour that had high negative loadings along Dimension 1. Browned and livery flavours were correlated with glycine, histidine, and proline. Glycine is an important compound in initial Maillard intermediates, which contribute to the formation of browned flavour in meat [53]. Proline has also been associated with browned flavour but is formed through the Amadori pathway [53]. In contrast, histidine plays a huge role in myoglobin structure and function. More specifically, distal histidine may interact with bound oxygen, which alters myoglobin structure and stability [54]. These changes are correlated with livery flavour, an off-flavour in beef [38]. Oxidised flavour was correlated with the presence of threonine, isoleucine, tyrosine, and phenylalanine. Interestingly, an increase of the aforementioned amino acids was shown to be associated with off-tastes such as acidity in dried meat products [55] and bitterness in some meat cuts [56].

The correlation biplot (Figure 6b) of PEF treated frozen lamb samples showed that livery and browned flavours were associated with flavour volatiles like dimethyl disulphide, 2-methyl-butanal, and heptanal, similar to chilled samples. Dipropyl disulphide and dipropyl trisulphide resulted in sharp rancid flavour and pungent smell in frozen pork dumplings [57]. Bueno, Resconi, Campo, Cacho, Ferreira and Escudero [27] further found that aldehydes like 2-methylpropanal, 3-methylbutanal, and 2-methylbutanal contributed negatively to lamb flavour because of their association with lipid oxidation [52], similar to present results. Ma, Hamid, Oey, Kantono, Faridnia, Yoo and Farouk [5] reported that browned and the livery flavours were correlated with PEF treated frozen-thawed shoulder cut on the 0 days of storage that had high concentrations of 2-heptanone and 2,3-octandione. Furthermore, oxidised flavour was mainly associated with 2-pentylfuran, and 2-ethyl-3-methyl pyrazine. Ma, Hamid, Oey, Kantono, Faridnia, Yoo and Farouk [5] also reported that PEF treated frozen-thawed shoulder cut of lamb was associated with 2-pentyl furan and 2-methylpropanal and 3-methylbutanal in loin cut stored for 7 days that were correlated with oxidised flavour.

A correlation biplot showing how fatty acids, amino acids, and volatile composition of PEF treated frozen lamb samples were correlated to sensory attributes is shown in Figure 6b. Meaty and juicy attributes had high positive loadings along Dimension 1. These attributes were mainly associated with the presence of volatiles, especially 2-nonanone, 2-pentylfuran, pyrrole, methyl pyrazine, 2-ethyl-3-methyl pyrazine, and thiophene, instead of fatty acids. This is because the principal reaction in frozen raw and cooked meat is lipid oxidation that releases volatiles that contribute to flavour [37]. Caporaso, Sink, Dimick, Mussinan and Sanderson [36] identified 2-nonanone as one the volatiles that contributed positively and significantly to the flavour of lamb, while pyrazine volatiles have been associated with roasted meat odour in lamb meat [27].

Browned and livery had high negative loadings along Dimension 1 and were associated with flavour volatiles like heptanal, 2-ethylfuran, pyridine, dimethyl disulphide, dimethyl trisulphide, and 3,5-diethyl-2-methyl pyrazine. Heptanal is of importance in cooked beef flavour and may impart a pleasant fruity flavour contributing towards the perception of browned flavour [12]. Additionally, 2-ethylfuran are formed from the degradation of lipids; although they are sometimes regarded as markers of lipid oxidation, they often contribute towards what is described as burnt, sweet, coffeelike flavour associated with browned flavour [58]. In contrast, dimethyl disulphide and dimethyl trisulphide are often described as sulphuric and unpleasant off flavours [59] which may explain the increased perception of livery flavours in beef [60].

Oxidised had high positive loadings along Dimension 2, and was mainly associated with the presence of amino acids such as threonine, phenylalanine, isoleucine, tyrosine, and methionine. Aromatic amino acids (phenylalanine, tyrosine, tryptophan, and histidine), especially tryptophan, showed high levels of oxidation that can contribute to oxidation flavour in dry-cured hams [61]. In addition, methionine can be easily oxidised to methional or methanethiol and has been found to be associated with off-odour and oxidised flavours in meat [62].

## 4. Conclusions

In this study, it is obvious that PEF significantly affected volatile composition and sensory characteristics of the seven lamb meat cuts. Temporal dominance of sensations (TDS) results showed that both storage and PEF treatments affected the temporal flavour profiles of meaty and oxidized flavour attributes. A longer storage period was associated with oxidized flavour, while PEF treatments for all chilled and frozen cuts were associated with browned and livery flavour attributes. Furthermore, PEF processing contributed to more oxidized flavour in PEF treated frozen–thawed rib cut stored for 7 days. PLSR revealed that with PEF treatment of chilled samples, meaty and juicy flavours were associated with the presence of fatty acids such as C18:0, SFA, 20:5(n-3), and n-3. In contrast, with PEF treatment of frozen meat, meaty and juicy flavours were associated with some volatile compounds especially 2-nonanone, 2-pentylfuran, pyrrole, methyl pyrazine, 2-ethyl-3-methyl pyrazine, and thiophene. Livery and browned flavours were associated with amino acids especially threonine, phenylalanine, isoleucine, tyrosine, methionine, and some volatile compounds, namely, heptanal, 2-ethylfuran, pyridine, dimethyl disulphide, dimethyl trisulphide, and 3,5-diethyl-2-methyl pyrazine in both PEF treated chilled and frozen lamb meat. These results imply that type of sample pre-treatments (chilled or frozen-thawed) and cuts, as well as storage, are important factors to consider when applying PEF treatments to lamb meat. One of the limitations of this study was the limited number of animals used in the study. Future studies should consider including more animal samples to validate the results of this study.

## Supplementary Materials

The following are available online at https://www.mdpi.com/article/10.3390/foods10051148/s1, Supplementary Table S1: Description of sensory attributes used in the TDS trial; Supplementary Table S2. The volatile composition of chilled lamb with and without PEF treatments (mg/100 g dry meat) during 0 or 7 days of storage; Supplementary Table S3. The volatile composition of frozen-thawed lamb with and without PEF treatments (mg/100 g dry meat) during 0 or 7 days of storage; Supplementary Table S4. Variable of Importance (VIP) coefficients of chilled samples for different variables; Supplementary Table S5. Variable of Importance (VIP) coefficients of chilled samples for fatty acids, amino acids, volatiles together with sensory; Supplementary Table S6. Variable of Importance (VIP) coefficients of frozen samples for different variables; Supplementary Table S7. Variable of Importance (VIP) coefficients of frozen samples for fatty acids, amino acids, volatiles together with sensory.
