# Supplementary material for: Pulsed Electric Field (PEF) Processing of Chilled and Frozen-Thawed Lamb Meat Cuts: Relationships between Sensory Characteristics and Chemical Composition of Meat
Source: Foods. 2021 May 20;10(5):1148. doi: 10.3390/foods10051148 (PMC8161130; doi:10.3390/foods10051148)
Supplement: Supplementary file 1 [file foods-10-01148-s001.zip › foods-1155954-supplementary.pdf]

Supplementary Table S1: Description of sensory attributes used in the TDS trial

Attribute	Taste definition in lamb	Reference
Initial tenderness	Minimum force necessary (first bite) to bite the meat sample with incisors teeth	3,different cuts of lamb (tederloin,shoulder, leg) sous vide cooked and then grilled to an internal temperature of 70 °C
Meaty	Flavours and aromatics associated with boiled lean lamb meat	Lamb boiled at 100 °C for 10 min.
Browned	Flavours associated with meat that is cooked and charred on the outside.	Lamb cooked at 70 °C, and allowed to brown on each side for 10 min.
Juicy	Amount of water retained in cooked meat that contribute to succulence.	Different cuts of cooked lamb with varying levels of juiciness.
Livery	Taste associated with animal organs.	Lamb liver cooked at 70 °C for 10 min.
Oxidized/ warmed over flavour	Flavour of reheated meat	Cooked lamb that was refrigerated for at least 24 h before reheating.

Supplementary Table S2. The volatile composition of chilled lamb with and without PEF treatments (mg/100 g dry meat) during 0 or 7 days of storage

No	Volatiles	Storage	Control							PEF						
			Knuckle	Loin	Rump	Rib	Shank	Shoulder	Topside	Knuckle	Loin	Rump	Rib	Shank	Shoulder	Topside
**Alcohols**																
1	1-Hexanol	0	0.681aA	0.612aB	0.636ayAB	0.643bAB	0.438byC	0.427ayC	0.599yB	0.526byC	0.477byD	0.495byCD	0.684axA	0.645ayB	0.407ayE	0.627B
		7	0.805A	0.675bB	0.828axA	0.675aB	0.713xB	0.495bxC	0.869axA	0.73xB	0.821axA	0.658bxC	0.489byD	0.702xBC	0.694axBC	0.662bC
**Ketones**																
2	2,3-Octanedione	0	0.951ayA	0.626ayCD	0.629ayCD	0.79ayB	0.667ayC	0.527ayE	0.553ayDE	0.349byB	0.238byD	0.476byA	0.47byA	0.483byA	0.349byB	0.284byC
		7	1.171axB	1.388axA	1.161axB	0.935axC	0.783axD	1.05axBC	1.043xBC	0.859bxBC	0.768bxC	0.923bxAB	0.59bxD	0.573bxD	0.613bxD	1.002xA
3	2-Heptanone	0	0.372byC	0.437BC	0.47byB	0.956xA	0.279bxD	0.374bxC	0.292byD	0.557ayC	0.468yD	0.585ayC	0.856xB	0.827axB	1.017ayA	0.456axD
		7	0.755xA	0.475bC	0.659bxB	0.345bxE	0.508yC	0.624byB	0.43bD	0.665xD	0.753axC	1.099axB	0.513ayE	0.527yE	1.173axA	0.782axC
4	2-Nonanone	0	1.779axB	1.541axC	2.117axA	1.663axBC	1.451aC	1.691axBC	0.812axD	0.309bxB	0.213bxCD	0.138bxE	0.621bxA	0.251bxBC	0.17bxDE	0.228bxCD
		7	0.369ayC	0.818ayB	1.477ayA	0.189ayD	1.233aA	1.364ayA	0.228ayC	0.085byA	0.047byCDE	0.061byBC	0.074byAB	0.04byDE	0.053byCD	0.033byE
**Aldehydes**																
5	ropanal, 2-meth	0	0.121byF	0.586ayB	0.715ayA	0.323byC	0.208byE	0.275byD	0.238xDE	0.249ayE	0.323byD	0.403byC	0.512aB	0.546aB	0.699aA	0.204yE
		7	0.243bxF	0.769axB	1.127axA	0.642axC	0.409bxDE	0.377bxE	0.484byD	0.531axD	0.622bxC	0.779bxA	0.387bF	0.442aE	0.731aB	0.555axD
6	utanal, 3-methy	0	0.243byF	1.054bA	0.956byB	0.649bxC	0.302byEF	0.354bxE	0.486bxD	1.429ayE	2.269axB	2.035aC	2.602axA	1.966axC	1.78axD	1.003ayF
		7	0.99bxC	1.426B	1.991xA	0.161byE	0.779bxCD	0.805byCD	0.617byD	1.977axB	1.368yC	1.849B	1.414ayC	1.548ayC	1.551ayC	2.24axA
7	utanal, 2-methy	0	0.281byF	1.254aB	1.412ayA	0.948ayC	0.374byE	0.357byEF	0.514ayD	0.46ayBC	0.19byE	0.471byBC	0.359bxD	0.564axA	0.485ayB	0.436bxC
		7	1.535axB	1.554B	2.521axA	0.181xD	0.914axC	0.861axC	0.805axC	0.935bxB	1.274xA	0.843bxB	0.199yD	0.149byD	0.563bxC	0.404byC
8	Hexanal	0	0.163bC	0.431yA	0.199byC	0.184byC	0.131byD	0.174byC	0.245yB	0.544ayB	0.39byC	0.717ayA	0.57aB	0.371ayC	0.411ayC	0.274yD
		7	0.190bD	2.618xA	1.24bxB	0.283bxCD	0.514bxC	0.413bxC	0.463bxC	2.298axB	2.364xB	1.711axC	0.595aF	2.958axA	1.143axE	1.274axD
9	Heptanal	0	0.544A	0.407aB	0.347bxC	0.402bxB	0.268byD	0.36bBC	0.173bE	0.583yB	0.38ayD	0.726ayA	0.51ayC	0.537ayBC	0.565ayBC	0.368ayD
		7	0.542bA	0.393bBC	0.291byD	0.582ayA	0.404axB	0.357bC	0.175bE	2.683axAB	2.985axA	2.237axABC	0.558axD	1.734axBC	1.301axCD	1.138axCD
10	Benzaldehyde	0	1.582ayD	3.058axA	2.622ayB	2.936aA	1.209ayE	1.46ayD	1.874ayC	0.162bC	0.854byA	0.926bxA	0.268bxB	0.16byC	0.172byC	0.109byC
		7	2.31axC	1.993byD	3.246axA	3.206aA	1.882xD	2.218axC	2.91axB	0.143bE	4.407axA	0.507byD	0.121byE	1.988xB	0.251bxE	1.328bxC
11	Nonanal	0	1.014ayAB	1.129aAB	0.959ayBC	0.62ayD	0.756ayCD	1.195aA	1.067aAB	0.304bxB	0.272bBC	0.224bD	0.665axA	0.263byC	0.298byB	0.238bCD
		7	2.36axA	1.242aB	1.069axC	1.237axB	1.138axBC	1.057aC	1.326aB	0.171byE	0.239bCD	0.238bCD	0.265byC	0.367bxB	0.417bxA	0.225bD
**Furans**																
12	2-Ethylfuran	0	0.049byE	0.085byD	0.157byC	0.262byA	0.254bxA	0.131byC	0.193bB	1.433ayBC	1.286ayC	1.565ayB	1.979axA	1.375aC	1.587ayB	0.818ayD
		7	0.127bxC	0.187bxB	0.217bxB	0.359bxA	0.147byC	0.138bxC	0.208bB	2.215axB	3.056axA	2.294axB	1.095ayE	1.168aE	1.817axC	1.439axD
13	2-Vinylfuran	0	0.392byBC	0.372byBC	4.082aA	0.467byB	0.538byB	0.226byC	0.532byB	0.92aB	0.638ayD	0.629byD	1.893axA	0.696ayCD	0.816ayBC	0.678ayCD
		7	2.289axC	2.854axB	4.103aA	2.537axBC	1.086axD	0.494bxE	0.863axDE	1.012bBC	1.105bxA	0.829bxE	0.89byDE	1.075axAB	1.062axAB	0.94axCD
14	2-Pentylfuran	0	0.51ayD	0.905axAB	0.845aB	0.925axA	0.463ayD	0.609ayC	0.469ayD	0.239bC	0.347bxB	0.501bxA	0.218bxCD	0.209byD	0.13byE	0.197byD
		7	0.946axA	0.651ayD	0.86aB	0.341ayE	0.738axC	0.726axC	0.712axCD	0.286bC	0.164byE	0.209byD	0.133byE	0.48bxA	0.202bxD	0.343bxB

No	Volatiles	Storage	Control							PEF						
			Knuckle	Loin	Rump	Rib	Shank	Shoulder	Topside	Knuckle	Loin	Rump	Rib	Shank	Shoulder	Topside
**Nitrogen and sulfur compounds**																
15	Pyridine	0	0.023byD	0.001byD	0.107byC	0.264bxB	0.383byA	0.135byC	0.269byB	2.764ayB	3.19ayA	2.455ayC	3.428aA	3.225ayA	3.202ayA	3.351ayA
		7	0.382bxC	0.009bxE	0.141bxD	0.027byE	0.402bxC	1.449bxB	1.544bxA	4.185axBC	4.472axA	4.1axC	3.821aD	4.386axAB	4.094axC	4.052axCD
16	Pyrrole	0	0.593ayA	0.309ayC	0.257ayD	0.252byD	0.241byD	0.248ayD	0.365ayB	0.349bxC	0.155bxDE	0.217axD	0.775axA	0.433axB	0.155byDE	0.147byE
		7	3.664axCD	8.259axA	3.388axD	7.016axB	3.733axC	2.612axE	3.314axD	0.04byA	0.025byBC	0.039byA	0.029byABC	0.041byA	0.036bxAB	0.022bxC
17	Pyrazine, methyl	0	0.022byE	0.237byBC	0.167byD	0.339byA	0.273byB	0.227byC	0.13byD	0.796ayC	1.082ayB	0.895ayC	0.623ayD	1.39ayA	0.323ayE	0.37ayE
		7	1.054bxF	2.254bxC	2.661bxB	1.107bxEF	3.141bxA	1.371xD	1.286bxDE	1.691axC	2.637axB	4.295axA	1.41axC	4.572axA	1.545xC	1.542axC
18	zine, 2-ethyl-3-m	0	0.656byB	0.101byE	0.653byB	0.546yC	0.202byD	0.92byA	0.496byC	2.471ayB	3.731ayA	2.353ayB	0.557yE	1.004ayD	1.375ayC	0.614ayE
		7	8.326bxC	7.212axD	9.197bxB	9.736bxA	1.328bxE	1.546bxE	8.49bxC	11.591axC	7.177axD	12.735axB	13.67axA	6.398axE	6.601axDE	11.971axC
19	ne, 3-ethyl-2,5-di	0	2.749byB	0.982byD	2.837byB	1.95byC	1.062byD	3.334byA	1.883yC	9.568ayB	16.503aA	8.643ayB	5.827ayC	4.993ayCD	4.484ayD	1.778yE
		7	9.89bxB	10.195bxB	17.53bxA	2.586bxE	5.395bxD	5.617bxD	8.504bxC	22.001axB	16.752aD	24.907axA	24.319axA	19.625axC	19.877axC	14.712axE
20	ne, 3,5-diethyl-2-ı	0	2.288byB	1.298bD	1.984byC	3.028bA	0.548byF	0.369byG	1.001byE	9.247ayC	1.998ayE	8.57ayC	6.397aD	22.388axA	17.145aB	6.337axD
		7	2.776bxB	1.322bD	3.54bxA	3.066bB	1.797axC	1.821bxC	2.005bxC	30.345axA	27.654axA	24.561axB	6.931aD	1.873ayE	17.503aC	3.947ayE
21	Thiophene	0	1.794ayBC	1.645ayCD	2.163ayA	1.895ayB	1.564yDE	1.404yE	2.109ayA	0.81byD	0.463byF	1.046byC	0.645byE	1.647xA	1.288yB	0.718byDE
		7	2.86xA	2.61xB	2.996axA	2.994axA	2.293axC	2.22axC	2.618axB	2.674xB	2.842xA	2.696bxB	0.97bxD	0.762byE	1.825bxC	1.001bxD
22	Dimethyl disulfid	0	0.56bA	0.512byBCD	0.498byCD	0.556byAB	0.544byAB	0.518byABC	0.471byD	2.177ayB	1.597ayD	1.897ayC	2.547axA	1.767ayC	1.795ayC	1.859ayC
		7	0.649bAB	0.611bxB	0.637bxAB	0.686bxAB	0.691bxAB	0.689bxA	0.705bxA	3.25axA	3.179axA	2.763axC	2.268ayE	2.995axB	2.608axD	2.538axD
23	Furanmethaneth	0	5.013ayD	14.492aB	17.585ayA	13.074aC	1.785ayE	1.311ayE	13.016ayC	0.483byCD	0.605bB	0.441byE	0.65bxA	0.457byDE	0.503byC	0.475byCDE
		7	15.329axB	14.704aB	20.533axA	13.516aC	12.208axD	13.249axC	9.642axE	0.568bxB	0.663bA	0.559bxB	0.572byB	0.677bxA	0.591bxB	0.562bxB
24	Dimethyl trisulfid	0	2.346byD	2.601aC	2.303bD	2.606yC	3.212bxA	2.811bxB	2.028bxE	3.737ayC	0.709byE	3.212ayCD	2.616D	12.216ayA	10.174ayB	2.575ayD
		7	2.716bxA	2.167bB	2.189bB	2.878xA	1.826byC	2.143byB	1.828byC	16.358axB	19.443axA	15.505axB	2.821D	13.022axC	12.228axC	3.137axD

a,b means with different letters in row show significant effect of processing in each cut; x,y means with different letters in column show significant effect of storage in each cut in same processing; A,B,C,D,E,F means with different letters in row show significant effect of cut in each processing using Fisher's least significant difference .

Supplementary Table S3. The volatile composition of frozen-thawed lamb with and without PEF treatments (mg/100 g dry meat) during 0 or 7 days of storage

No	Volatiles	Storage	Control							PEF						
			Knuckle	Loin	Rump	Rib	Shank	Shoulder	Topside	Knuckle	Loin	Rump	Rib	Shank	Shoulder	Topside
**Alcohols**																
1	1-Hexanol	0	0.925yA	0.714ayBC	0.948ayA	0.826yAB	0.61byC	0.6yC	0.83ayAB	1.453A	0.509byC	0.702byBC	0.976yB	0.657ayC	0.663C	0.529byC
		7	1.249xB	1.914axA	1.443xB	1.225bxB	1.364xB	1.366axB	1.42xB	1.386AB	0.755bxC	1.508xA	1.393axAB	1.309xB	0.707bC	1.417xAB
**Ketones**																
2	2,3-Octanedione	0	2.37byB	0.844axC	0.952byC	3.022ayA	3.182ayA	0.939ayC	0.237byD	5.072aA	0.674byE	1.562ayBC	1.815byB	1.209byCD	0.951ayDE	0.801ayDE
		7	3.464bxBC	0.263byE	3.153bxC	4.165bxAB	5.022xA	2.059bxD	5.169axA	4.704aC	2.959axE	6.177axB	10.805axA	4.71xC	3.484axDE	4.479bxCD
3	2-Heptanone	0	0.165byD	0.22yD	1.072ayA	0.225byD	0.835aB	0.424yC	0.464ayC	1.708ayA	0.267yC	0.576byB	0.569ayB	0.486byBC	0.413yBC	0.299byC
		7	1.031bxBC	1.17xB	1.538bxA	0.499bxE	0.773bD	0.541bxE	0.971bxC	3.588axBC	1.072xE	5.027axA	3.971axAB	2.481axCD	1.548axDE	3.198axBC
4	2-Nonanone	0	1.257axC	0.706ayD	2.838aA	0.519ayD	1.952axB	1.144aC	0.623ayD	0.061b	0.068bx	0.066by	0.082by	0.066by	0.082by	0.073b
		7	0.955ayDE	3.614axA	2.484aB	0.729axE	1.359ayC	1.127aCD	1.004axDE	0.188bAB	0.047byD	0.184bxBC	0.258bxAB	0.117bxBC	0.198bxAB	0.098bC
**Aldehydes**																
5	ropanal, 2-meth	0	2.542axA	0.314ayC	0.814ayB	0.52yBC	0.327yC	0.263yC	0.221yC	1.091byA	0.23byC	0.457byB	0.441yB	0.443yB	0.318yBC	0.209yC
		7	3.031axB	4.478axA	2.959xB	5.194xA	1.885xC	1.902axC	2.806axB	1.249byA	0.818bxC	4.387xA	4.094xA	2.594xB	1.575bxBC	0.727bxBC
6	utanal, 3-methy	0	0.585byD	0.675yCD	1.005byA	0.31byE	0.836byB	0.746BC	0.763byBC	2.005ayB	0.76yD	2.097ayB	1.182ayC	1.207ayC	0.911yCD	5.442ayA
		7	6.501xB	9.136axA	6.744xB	6.89xB	4.021xC	4.344aC	9.511axA	5.062xABC	1.931bxD	6.796xAB	7.028xA	5.333xABC	3.637bxCD	4.567bxBC
7	utanal, 2-methy	0	0.448byDE	0.816ayC	2.135ayB	0.191ayE	0.759ayCD	0.722aCD	6.737ayA	0.768ayA	0.114byD	0.738byA	0.292ayBC	0.228byCD	0.384byB	0.185byCD
		7	8.075axCD	10.819axB	9.018axC	6.725axDE	5.02axF	5.346aEF	13.477axA	1.787bxAB	0.769bxC	2.073bxA	1.379bxB	0.605bxC	0.668bxC	1.452bxB
8	Hexanal	0	0.132byD	0.168byD	0.441byA	0.149byD	0.303bC	0.186ybD	0.374yB	2.422ayA	0.556ayC	1.753ayB	0.661ayC	0.504ayC	0.679ayC	0.535yC
		7	0.676bxA	0.346bxC	0.576bxB	0.293bxC	0.374bC	0.33xbC	0.743bxA	5.256axA	1.969axB	5.574axA	4.524axA	1.598axB	1.711axB	4.439axA
9	Heptanal	0	0.201byC	0.187bC	0.268bBC	0.314byAB	0.388xA	0.269bBC	0.184byC	2.433ayA	0.517ayBC	2.254ayA	0.611ayBC	0.527yBC	0.864ayB	0.513ayC
		7	0.336bxAB	0.271bBC	0.336bAB	0.205bxC	0.279byBC	0.117bD	0.404bxA	5.289axAB	1.818axC	6.169axA	4.213axB	1.616axC	1.895axC	4.459axB
10	Benzaldehyde	0	0.968yEF	1.196yDE	3.044ayB	0.841ayF	1.538ayC	1.456ayCD	4.131ayA	1.282A	1.31yA	0.438byBC	0.528byB	0.36byCD	0.226byD	0.314byCD
		7	5.024axB	5.844axA	4.327axBC	4.336xbBC	3.312axD	3.997axCD	6.159axA	1.135bB	1.925bxB	1.117bxB	10.116axA	0.852bxB	0.544bxB	1.388bxB
11	Nonanal	0	0.77yBC	0.692ayC	0.699ayC	0.922ayB	1.226aA	0.922aB	0.728ayBC	0.691A	0.082byDE	0.153byD	0.591bB	0.41bC	0.084byDE	0.045byE
		7	3.279axA	2.234axB	1.96axBC	1.734axC	1.33aD	1.324aD	3.631axA	0.719bB	0.262bxD	0.627bxB	1.971aA	0.407bC	0.17bxD	0.46bxC
**Furans**																
12	2-Ethylfuran	0	3.162xA	0.444bxB	0.151byBC	0.31byBC	0.231byBC	0.286byC	0.321byC	4.051A	0.835ayB	1.836ayB	0.833ayC	1.203ayCD	1.734ayCD	0.661ayD
		7	2.383byA	0.124byB	0.553bxB	0.12bxB	0.479bxC	0.147bxC	0.52bxC	4.419aA	1.82axB	4.107axA	4.048axA	2.428axB	2.587axB	4.412axA
13	2-Vinylfuran	0	0.526bxD	3.993axB	0.274byDE	2.162axC	0.02byE	0.697yD	4.504ayA	1.483aA	0.51byEF	0.857ayD	1.3bB	0.981ayC	0.568yE	0.443byF
		7	0.226byF	1.043yE	5.778axA	0.917byE	2.457axD	3.037axC	5.238axB	1.952aB	0.951xBC	2.05bxB	4.044aA	1.262bxBC	0.7bxC	1.473bxBC
14	2-Pentylfuran	0	0.241ayD	0.282ayD	1.133ayA	0.277byD	0.914aB	0.531ayC	0.154byD	0.08bE	0.171bxD	0.522bxA	0.427ayBC	0.442bB	0.207bD	0.365ayC
		7	1.761axAB	1.609axB	2.029axA	0.714axC	0.991aC	0.848axC	1.646axB	0.088bD	0.084byD	0.235byC	0.197bxC	0.404bA	0.206bBC	0.29bxD

No	Volatiles	Storage	Control							PEF						
			Knuckle	Loin	Rump	Rib	Shank	Shoulder	Topside	Knuckle	Loin	Rump	Rib	Shank	Shoulder	Topside
**Nitrogen and sulfur compounds**																
15	Pyridine	0	0.032bAB	0.037bA	0.028byBC	0.032byAB	0.011byD	0.025bC	0.032bAB	6.116ayA	3.482ayDE	4.47ayC	5.202aB	3.724ayD	3.86aCD	2.966ayE
		7	0.031bC	0.03bC	0.046bxB	0.082bxA	0.075bxA	0.02bD	0.035bC	7.21axAB	3.852axD	8.255axAB	9.105aA	4.265axCD	3.512aD	6.087axBC
16	Pyrrole	0	0.725axA	0.232ayD	0.409ayB	0.418ayB	0.374ayBC	0.306ayCD	0.429ayB	0.032byB	0.021byE	0.024byCDE	0.053byA	0.025byCD	0.027byC	0.023byDE
		7	0.596ayCD	0.92axA	0.676axC	0.517axD	0.679axC	0.501axD	0.807axB	0.049bxB	0.038bxC	0.033bxDE	0.063bxA	0.033bxD	0.036bxCD	0.03bxE
17	Pyrazine, methyl	0	0.171yD	1.239ayB	1.245ayB	0.142byD	0.685ayC	1.197ayB	4.538ayA	0.367yA	0.262byD	0.315bBC	0.346aAB	0.321byABC	0.286byCD	0.169byE
		7	12.076axA	8.368axB	7.029axBC	3.949axE	5.139axDE	6.653axCD	12.099axA	0.68bxAB	0.595bxAB	0.29bB	0.989bA	0.47bxB	0.593bxAB	0.731bxAB
18	zine, 2-ethyl-3-m	0	2.541ayD	4.614ayBC	4.396ayC	1.088ayE	3.253ayD	5.298aB	9.425ayA	0.969byB	0.52byBC	2.138byA	0.19byD	0.993bB	2.09byA	0.419byCD
		7	22.308axA	20.521axAB	17.037axC	7.923axD	16.642axC	4.399aE	19.442axB	2.487bxA	1.03bxB	3.135bxA	2.649bxA	0.711bB	1.066bxB	2.294bxA
19	ne, 3-ethyl-2,5-di	0	8.165ayCD	11.378ayB	14.495ayA	2.006yE	6.804ayD	9.796ayBC	14.277ayA	2.247byC	1.44byC	6.72byA	1.877C	3.506bB	7.166bA	1.539byC
		7	32.412axD	36.442axC	40.596axB	8.933xE	35.015axCD	7.524aE	45.761axA	9.317bxA	2.321bxB	9.277bxA	8.155A	2.523bB	3.901bxB	6.861bxA
20	ne, 3,5-diethyl-2-r	0	2.466byB	1.179byCD	1.36byC	3.187byA	0.997byD	1.287byCD	2.354byB	10.374A	3.234aB	11.49aA	5.014ayB	10.573axA	9.35ayA	4.881ayB
		7	3.487xCD	6.507axA	4.388bxBC	4.154bxBC	3.082xD	3.91bxBCD	4.443bxB	8.326aCD	2.811bE	11.799aAB	9.778axBC	2.98yE	6.594axD	13.446axA
21	Thiophene	0	3.198ayAB	3.081ayAB	2.442ayC	3.559aA	2.461ayC	2.72yBC	2.466ayB	2.145bB	1.019byC	1.804bB	1.149bC	1.113bC	2.709yA	0.933byC
		7	5.149axAB	4.899axBC	5.77axA	4.276B	5.329axAB	4.772axBC	4.279axC	2.107bAB	1.238bxC	2.227bAB	2.696A	1.255bC	1.574bxBC	2.041bxABC
22	Dimethyl disulfid	0	0.726bB	0.596bC	0.433byD	0.729byB	0.803byA	0.711byB	0.583byC	4.65ayA	2.412ayE	3.338ayC	3.985ayB	2.963ayCD	2.528ayE	2.647ayDE
		7	0.7bC	0.674bC	0.698bxC	0.822bxAB	0.876bxA	0.78bxABC	0.722bxBC	5.775axB	3.814axC	6.12axAB	6.956axA	3.943axC	3.584axC	6.053axB
23	Furanmethaneth	0	2.214ayD	9.435ayA	10.463ayA	2.243ayD	7.419ayB	4.586aC	2.492ayD	0.824bA	0.72byB	0.614byC	0.838bA	0.717bB	0.692byB	0.639byC
		7	42.545axAB	36.899axC	39.409axBC	7.972axD	36.192axC	4.146aE	44.416axA	0.861bBC	0.971bxAB	0.804bxBC	1.106bA	0.798bC	0.825bxBC	0.901bxBC
24	Dimethyl trisulfid	0	3.553bAB	2.428bBC	2.329byBC	4.528aA	2.06byC	2.295bBC	2.943aBC	11.757aAB	2.768ayC	7.885aB	2.069byC	9.58aAB	12.031ayA	3.217ayC
		7	2.437bD	2.6D	3.048bxBCD	4.321bA	3.257bxBC	2.847bCD	3.511bB	9.245aB	3.533xC	10.398aB	11.011axAB	4.34aC	8.315axB	14.353axA

[a,b] means with different letters in row show significant effect of processing in each cut; [x,y] means with different letters in column show significant effect of storage in each cut in same processing; [A,B,C,D,E,F] means with different letters in row show significant effect of cut in each processing using Fisher's least significant difference .

Supplementary Table S4. Variable of Importance (VIP) coefficients of chilled samples for different variables

Fatty acids	VIP coefficient	Amino acids	VIP coefficient	Volatiles	VIP coefficient
20:2 (n-6)	1.672	HIS	1.256	VOC15	1.466
MUFA	1.338	PRO	1.163	VOC22	1.433
18:1 cis-9	1.326	THR	1.153	VOC16	1.328
C20:0	1.280	ILE	1.120	VOC9	1.225
C14:0	1.228	TYR	1.067	VOC17	1.170
C24:0	1.193	GLY	1.033	VOC8	1.154
C16:1	1.176	PHE	1.031	VOC21	1.132
PUFA	1.064	GLU	1.000	VOC7	1.106
n-6	1.015	ASP	0.990	VOC14	1.090
SFA	0.983	MET	0.984	VOC24	1.063
n-3	0.982	VAL	0.961	VOC12	1.044
18:2 (n-6)	0.960	ALA	0.928	VOC18	1.043
20:1 (n-9)	0.954	LEU	0.881	VOC11	1.016
18:3 (n-3)	0.898	TRP	0.867	VOC20	0.994
20:3 (n-6)	0.862	LYS	0.819	VOC4	0.942
C18:0	0.835	SER	0.818	VOC23	0.886
20:5 (n-3)	0.728	ORN	0.787	VOC19	0.872
C21:0	0.713			VOC3	0.767
C17:0	0.701			VOC10	0.718
P:S	0.633			VOC13	0.683
C22:0	0.593			VOC2	0.576
C16:0	0.495			VOC5	0.554
n-6/n-3	0.010			VOC1	0.323
				VOC6	0.255

Supplementary Table S5. Variable of Importance (VIP) coefficients of chilled samples for fatty acids, amino acids, volatiles together with sensory

Variable	VIP coefficient
VOC22	2.148
VOC15	2.138
VOC9	1.900
VOC8	1.869
VOC12	1.654
VOC16	1.651
HIS	1.607
VOC24	1.493
VOC3	1.458
PRO	1.393
VOC20	1.356
VOC2	1.353
VOC21	1.330
GLY	1.317
VOC17	1.244
VOC14	1.230
GLU	1.220
TRP	1.213
VOC4	1.206
ILE	1.166
TYR	1.165
VOC7	1.140
THR	1.129
MET	1.098
VOC18	1.082
PHE	1.057
ASP	1.001
VOC11	0.970
20:1 (n-9)	0.935
VOC23	0.851
ALA	0.835
VAL	0.831
VOC19	0.804
ORN	0.758
LEU	0.749
C20:0	0.705
SER	0.693
VOC13	0.611
LYS	0.594
C14:0	0.532
VOC10	0.435

C24:0	0.349
C22:0	0.330
VOC5	0.323
C16:1	0.312
n-3	0.307
C17:0	0.305
20:3 (n-6)	0.273
20:5 (n-3)	0.270

Supplementary Table S6. Variable of Importance (VIP) coefficients of frozen samples for different variables

Fatty acids	VIP coefficient	Amino acids	VIP coefficient	Volatiles	VIP coefficient
n-3	1.378	ORN	1.239	VOC12	1.541
18:3 (n-3)	1.317	ASP	1.169	VOC22	1.497
PUFA	1.315	SER	1.168	VOC4	1.490
n-6	1.249	PHE	1.087	VOC15	1.421
18:2 (n-6)	1.237	HIS	1.061	VOC19	1.270
20:3 (n-6)	1.234	GLY	1.042	VOC9	1.250
20:2 (n-6)	1.229	THR	1.033	VOC18	1.235
C18:0	1.086	ILE	1.002	VOC20	1.214
20:5 (n-3)	1.066	LEU	0.984	VOC14	1.186
SFA	1.057	VAL	0.970	VOC3	1.096
20:1 (n-9)	1.049	LYS	0.943	VOC24	1.079
MUFA	0.942	TRP	0.930	VOC23	1.053
18:1 cis-9	0.941	PRO	0.901	VOC6	1.036
C22:0	0.910	GLU	0.879	VOC17	0.882
C16:0	0.867	MET	0.844	VOC8	0.807
C16:1	0.862	TYR	0.829	VOC11	0.722
C24:0	0.779	ALA	0.789	VOC5	0.661
C14:0	0.766			VOC1	0.519
C17:0	0.764			VOC10	0.518
C21:0	0.657			VOC7	0.254
C20:0	0.542			VOC21	0.099
P:S	0.537			VOC16	0.080
n-6/n-3	0.318			VOC13	0.068
				VOC2	0.046

Supplementary Table S7. Variable of Importance (VIP) coefficients of frozen samples for fatty acids, amino acids, volatiles together with sensory

Variable	VIP coefficient
n-3	1.529
18:3 (n-3)	1.454
PUFA	1.442
VOC4	1.371
n-6	1.361
20:3 (n-6)	1.354
18:2 (n-6)	1.349
20:2 (n-6)	1.333
VOC18	1.320
VOC12	1.273
VOC22	1.267
ORN	1.261
C18:0	1.231
VOC19	1.226
VOC15	1.201
20:5 (n-3)	1.200
SFA	1.195
SER	1.190
ASP	1.172
VOC9	1.125
PHE	1.110
20:1 (n-9)	1.108
HIS	1.062
VOC20	1.030
THR	1.023
ILE	1.017
GLY	1.012
MUFA	1.006
18:1 cis-9	1.006
LEU	0.989
C22:0	0.987
VAL	0.981
C16:0	0.977
VOC24	0.948
VOC3	0.948
VOC17	0.944
VOC14	0.941
LYS	0.939
TRP	0.932
C16:1	0.907
PRO	0.904
C24:0	0.885
C17:0	0.879
C14:0	0.874
GLU	0.870
VOC6	0.864
MET	0.829
TYR	0.820
VOC8	0.782
VOC23	0.767
ALA	0.758

VOC5	0.733
C21:0	0.691
VOC1	0.669
C20:0	0.608
P:S	0.545
VOC11	0.463
n-6/n-3	0.407
VOC21	0.304
VOC10	0.291
VOC2	0.280
VOC16	0.134
VOC7	0.108
VOC13	0.067
